# Modern psychometrics applied in rheumatology–A systematic review

**DOI:** 10.1186/1471-2474-13-216

**Published:** 2012-10-31

**Authors:** Liseth Siemons, Peter M ten Klooster, Erik Taal, Cees AW Glas, Mart AFJ Van de Laar

**Affiliations:** 1 Department of Psychology, Health & Technology, Arthritis Center Twente, University of Twente, PO Box 217, Enschede, 7500 AE, the Netherlands; 2 Department of Research Methodology, Measurement and Data Analysis, Arthritis Center Twente, University of Twente, Enschede, The Netherlands; 3 Department of Rheumatology, Medisch Spectrum Twente, Arthritis Center Twente, Enschede, The Netherlands

**Keywords:** Clinical measures, Item response theory, Modern psychometrics, Patient-reported outcomes, Rheumatology

## Abstract

**Background:**

Although item response theory (IRT) appears to be increasingly used within health care research in general, a comprehensive overview of the frequency and characteristics of IRT analyses within the rheumatic field is lacking. An overview of the use and application of IRT in rheumatology to date may give insight into future research directions and highlight new possibilities for the improvement of outcome assessment in rheumatic conditions. Therefore, this study systematically reviewed the application of IRT to patient-reported and clinical outcome measures in rheumatology.

**Methods:**

Literature searches in PubMed, Scopus and Web of Science resulted in 99 original English-language articles which used some form of IRT-based analysis of patient-reported or clinical outcome data in patients with a rheumatic condition. Both general study information and IRT-specific information were assessed.

**Results:**

Most studies used Rasch modeling for developing or evaluating new or existing patient-reported outcomes in rheumatoid arthritis or osteoarthritis patients. Outcomes of principle interest were physical functioning and quality of life. Since the last decade, IRT has also been applied to clinical measures more frequently. IRT was mostly used for evaluating model fit, unidimensionality and differential item functioning, the distribution of items and persons along the underlying scale, and reliability. Less frequently used IRT applications were the evaluation of local independence, the threshold ordering of items, and the measurement precision along the scale.

**Conclusion:**

IRT applications have markedly increased within rheumatology over the past decades. To date, IRT has primarily been applied to patient-reported outcomes, however, applications to clinical measures are gaining interest. Useful IRT applications not yet widely used within rheumatology include the cross-calibration of instrument scores and the development of computerized adaptive tests which may reduce the measurement burden for both the patient and the clinician. Also, the measurement precision of outcome measures along the scale was only evaluated occasionally. Performed IRT analyses should be adequately explained, justified, and reported. A global consensus about uniform guidelines should be reached concerning the minimum number of assumptions which should be met and best ways of testing these assumptions, in order to stimulate the quality appraisal of performed IRT analyses.

## Background

Since there is no gold standard for the assessment of disease severity and impact in most rheumatic conditions, it is common practice to administer multiple outcome measures to patients. Initially, the severity and impact of most rheumatic conditions was typically evaluated with clinical measures (CMs) [1,2] such as laboratory measures of inflammation like the erythrocyte sedimentation rate [3] and physician-based joint counts [4,5]. Since the eighties of the last century, however, rheumatologists have increasingly started to use patient-reported outcomes (PROs) [1,2]. As a result, a wide variety of PROs are currently in use, varying from single item visual analogue scales (e.g. pain or general health) to multiple item scales like the health assessment questionnaire (HAQ) [6] which measures a patient’s functional status and the 36-item short form health survey (SF-36) which measures eight dimensions of health related quality of life [7].

Statistical methods are essential for the development and evaluation of all outcome measures. By far, most health outcome measures have been developed using methods from classical test theory (CTT). In recent years, however, an increase in the use of statistical methods based on item response theory (IRT) can be observed in health status assessment [8-10]. Extensive and detailed descriptions of IRT can be found in the literature [11-14]. In short, IRT is a collection of probabilistic models, describing the relation between a patient’s response to a categorical question/item and the underlying construct being measured by the scale [11,15]. IRT supplements CTT methods, because it provides more detailed information on the item level and on the person level. This enables a more thorough evaluation of an instrument’s psychometric characteristics [15], including its measurement range and measurement precision. The evaluation of the contribution of individual items facilitates the identification of the most relevant, precise, and efficient items for the assessment of the construct being measured by the instrument. This is very useful for the development of new instruments, but also for improving existing instruments and developing alternate or short form versions of existing instruments [16]. Additionally, IRT methods are particularly suitable for equating different instruments intended to measure the same construct [17] and for cross-cultural validation purposes [18]. Finally, IRT provides the basis for developing item banks and patient-tailored computerized adaptive tests (CATs) [9,19,20].

Although IRT appears to be increasingly used within health care research in general, a comprehensive overview of the frequency and characteristics of IRT analyses within the rheumatic field is lacking. The Outcome Measures in Rheumatology (OMERACT) network recently initiated a special interest group aimed at promoting the use of IRT methods in rheumatology [21]. An overview of the use and application of IRT in rheumatology to date may give insight into future research directions and highlight new possibilities for the improvement of outcome assessment in rheumatic conditions. Therefore, the aim of this study was to systematically review the application of IRT to clinical and patient-reported outcome measures within rheumatology.

## Methods

### Search strategy

**Figure 1:**
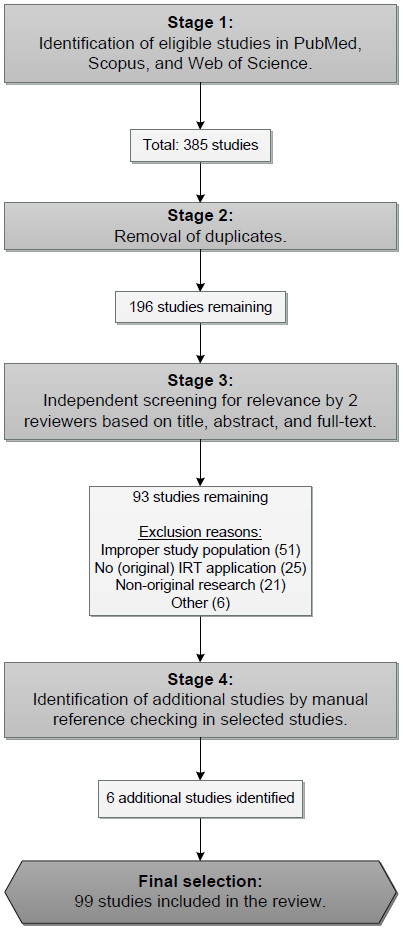
Flowchart of the search process.

Figure 1 presents an overview of the various stages followed during the search process, starting with an extensive literature search in April 2012 to identify all eligible studies up to and including the year 2011. Electronic database searches of PubMed, Scopus, and Web of Science were carried out, using the terms 'Item response theor*' OR 'Item response model*' OR 'latent trait theor*' OR Rasch OR Mokken, in combination with Rheumat* OR Arthros* OR arthrit*.

### Inclusion and exclusion criteria

Only original research articles written in English were included. Articles were considered original when they included original data and when they performed analyses on this data in order to achieve a defined study objective. To be included, studies should present an application of IRT in a sample of which at least 50% had some kind of rheumatic disease. In cases where less than 50% of the study sample consisted of rheumatic patients (i.e. inflammatory rheumatism, arthrosis, soft tissue rheumatism), the study was only included when the rheumatic sample was analysed separately from the rest of the sample. Reviews, letters, editorials, opinion papers, abstracts, posters, and purely descriptive studies were excluded. No limitations were set for study design.

### Study identification and selection

The search strategy resulted in a total of 385 studies. After the removal of 189 duplicates, 196 unique articles were identified. Two reviewers independently screened all 196 studies for relevance based on the abstract and title identified from the initial search. If no evident inclusion or exclusion reasons were identified, the full-text was examined. In total, 103 studies did not meet inclusion criteria and were excluded. The main reasons for exclusion were: the study population (i.e. the study population was not clearly defined or the study contained a rheumatic sample <50% of the total sample which was not separately analysed), the statistical analyses (i.e. no IRT application), and the article type (i.e. non-original research). Figure 1 includes an overview of the exclusion reasons followed by the number of articles removed.

### Data extraction

First, two reviewers independently evaluated a random sample of 15 articles. Both general study information as well as IRT-specific information were extracted, using a purpose-made checklist ( Additional file 1) based on both expert input and important issues as mentioned in Tennant and Conaghan [22], Reeve and Fayers [15], and Orlando [23]. Inter-rater agreement of the evaluated variables was moderate to high, with Cohen’s kappa ranging from 0.60 to 1.00. Most of the disagreements were caused by differing interpretations of some of the extracted variables. For instance, one of the reviewers interpreted the checklist on “performed analyses” as performed analyses using IRT based methods only, whereas the other reviewer interpreted it more broadly including classical test theory methods as well (the latter being the correct method). Consensus about these differences was reached by discussion. Next, one of these reviewers also evaluated the remaining 84 articles.

#### General study information

General information concerned the author(s), publication year, study population, the populations’ country of origin, total number of participants (N), study design of the IRT analyses (i.e. cross-sectional or longitudinal), type of outcome measure (PRO or CM), and main measurement intention (e.g. quality of life, pain, overall physical functioning).

#### Purpose of analyses

The purpose of the analyses was determined by the main goal the author(s) of the article pursued (e.g. the development, evaluation, comparison, or cross-cultural validation of instruments).

#### Specific IRT analyses

Before a researcher can perform IRT analyses, an appropriate IRT model should be selected. Unidimensional models are most widely applied, the simplest being the Rasch model which assumes that the items are equally discriminating and vary only in their difficulty. The 2-parameter logistic model (2-PL model) extends the Rasch model by assuming that the items have a varying ability to discriminate among people with different levels of the underlying construct [11,15,19,23]. These models can be specified further for polytomous items. The rating scale model, graded response model, modified graded response model, partial credit model, and generalized partial credit model can be applied in case of ordered categorical responses. The nominal response model can be applied when response categories are not necessarily ordered [11,15,19,23,24]. The rating scale model and the partial credit model are generalizations of the Rasch model, the other models are generalizations of the 2-PL model. In addition to these unidimensional models, multidimensional models and specific non-parametric models like the Mokken model [25,26] have been developed. Differences in model assumptions should be taken into account when choosing a model and model choice should be motivated by taking the discrimination equality of the items and the number of (ordered) response categories into consideration [15,22-24].

The applied IRT software and the corresponding item and person parameter estimation method(s) should also be cited, since not all software packages report the findings in the same way [22] and because the use of different estimation methods may result in different parameter estimates [11].

To make IRT results interpretable and trustworthy, three principal assumptions should be evaluated when applying a unidimensional IRT model [15,23]. The first assumption concerns unidimensionality, meaning that the set of test items measure only a single construct [11,15,22,23]. Analyses for checking the unidimensionality can include different types of factor analysis of the items or the residuals. A more advanced method would be to compare a unidimensional IRT model with a multidimensional IRT model, for instance using a likelihood ratio test. The second (related) assumption concerns local independence of the items. When this assumption is violated this may indicate that the items have more in common with each other than just the single underlying construct [11,15,22,23]. This may either point to response dependency (e.g. overlapping items in the scale) or to multidimensionality of the scale [22]. It can lead to biased parameter estimates and wrong decisions about, for instance, item selection [15]. Local independence can be tested by a factor analysis of the residual covariations, or with more specific statistics targeted at responses to pairs of items [12]. The third assumption concerns the model’s appropriateness to reflect the true relationship among the underlying construct and the item responses [11,15,22,23]. This can be examined with both item and person fit statistics. More information about these assumptions and suggestions about which aspects to report can be found in the literature [11,15,22,23].

Other useful IRT applications include the evaluation of the presence of differential item functioning, the reliability and measurement precision, the ordering of the response categories or item thresholds, and the hierarchical ordering and distribution of persons and items along the scale of the underlying construct.

Differential item functioning (DIF, also called item bias) is present when patients with similar levels of the underlying construct being measured respond differently to an item [15,22]. Commonly examined types of DIF are DIF across gender and age [22].

Global IRT reliability is equivalent to Cronbach’s alpha, with the difference that not the raw score but the IRT score is being used in its calculation. Which specific global reliability statistics are presented usually depends on the software package used. Contrary to CTT methods, IRT also provides information about the local reliability [12] and, related to this, the instrument’s measurement precision along the scale of the underlying construct.

With rating scale analysis, the ordering of the response categories or item thresholds can be checked, enabling the evaluation of the appropriateness or redundancy of the response categories [15]. Likewise, the hierarchical ordering and/or distribution of persons and items along the scale can be analysed to determine the measurement range of the outcome measure and to determine whether the items function well for the included population sample or whether there is a mismatch between them [23].

## Results

### General information of included studies

**Figure 2:**
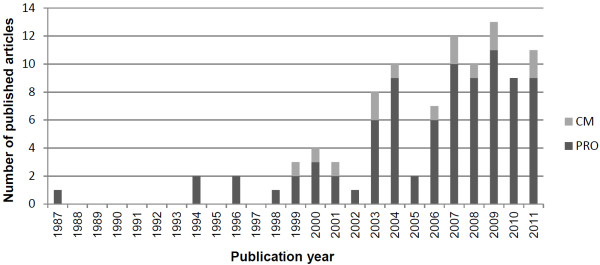
Number of published articles reporting the application of IRT within rheumatology.

The initial database search yielded a total of 93 eligible studies. Six additional studies were identified by manual reference checks of the selected studies. This resulted in a final selection of 99 studies ( Additional file 2). Figure 2 shows that the prevalence of IRT analysis within rheumatology increased markedly over the past decades. This is consistent with conclusions from Hays et al. [19], and with findings from Belvedere and Morton [8] who examined the frequency of Rasch analyses in the development of mobility instruments.

Table 1 presents an overview of the most prominent results. By far, most research was carried out with patients from the United States or the United Kingdom, but data from patients from the Netherlands and Canada were also regularly used. The vast majority of studies involved cross-sectional IRT analyses. It could also be observed that an increasing number of studies perform longitudinal IRT analyses since the 21st century, as represented by a rise of DIF testing over time.

**Table 1:** Overview of the most prominent results

**Variable**	**PRO-studies**		**CM-studies**		**Total-studies**	
	**n**	% *	**n**	% *	**n**	% *
US	26	30.6	4	28.6	30	30.3
UK	24	28.2	2	14.3	26	26.3
Netherlands	11	12.9	4	28.6	15	15.2
Canada	10	11.8	1	7.1	11	11.1
Other	32	37.6	5	35.7	37	37.4
Cross-sectional	76	89.4	14	100.0	90	90.9
Longitudinal	13	15.3	2	14.3	15	15.2
RA	43	50.6	5	35.7	48	48.5
OA	31	36.5	3	21.4	34	34.3
Other	31	36.5	7	50.0	38	38.4
Overall physical functioning	33	38.8	2	14.3	35	35.4
Quality of life	26	30.6	2	14.3	28	28.3
Specific functioning	10	11.8	0	0.0	10	10.1
Pain	7	8.2	0	0.0	7	7.1
Psychological constructs	3	3.5	0	0.0	3	3.0
Work disability	5	5.9	0	0.0	5	5.0
Disease activity	0	0.0	3	21.4	3	3.0
Disease damage or radiographic severity	0	0.0	3	21.4	3	3.0
Other	11	12.9	4	28.6	15	15.2
Development/evaluation new measures	25	29.4	2	14.3	27	31.4
Evaluation existing measures	31	36.5	6	42.9	37	37.4
Development/evaluation alternate/short form	11	12.9	2	14.3	13	13.1
Development item bank or CAT	4	4.7	0	0.0	4	4.0
Cross-cultural validation	7	8.2	2	14.3	9	9.1
Other	11	12.9	3	21.4	14	14.1
Bigsteps/Winsteps	28	32.9	3	21.4	31	31.3
RUMM	29	34.1	6	42.9	35	35.4
Other/not specified	29	34.1	5	35.7	34	34.3
Rasch	72	84.7	12	85.7	84	84.8
2-PLM	13	15.3	1	7.1	14	14.1
Mokken	3	3.5	1	7.1	4	4.0
Unidimensionality	65	76.5	10	71.4	75	75.8
Local independence	16	18.8	1	7.1	17	17.2
Appropriateness model (fit analyses)	77	90.6	13	92.9	90	90.9
DIF	50	58.8	6	42.9	56	56.6
Person/item separation/reliability	52	61.2	10	71.4	62	62.6
Hierarchical ordering/distribution of items/persons	57	67.1	9	64.3	66	66.7
Rating scale analysis	30	35.3	7	50.0	37	37.4
Measurement precision of the scale	10	11.8	1	7.1	11	11.1

PRO: patient-reported outcome (N=85), CM: clinical measure (N=14), RA: rheumatoid arthritis, OA: osteoarthritis, CAT: computerized adaptive test, IRT: item response theory, 2-PLM: 2 parameter logistic model, DIF: differential item functioning.

* Note that some studies can be assigned to multiple subcategories, therefore, the sum of the percentages within a category exceeds 100%.

Study samples varied from as little as 18 persons in the study of Penta et al. [27] to as many as 16519 persons in the study conducted by Wolfe et al. [28]. Most studies (92.9%) performed analyses on a population sample of at least 50 persons.

In 85 of the 99 studies IRT analyses were applied to PROs. The remaining 14 studies applied IRT to CMs. The vast majority of the studies applied IRT to data gathered from patients suffering from rheumatoid arthritis (RA) or osteoarthritis (OA).

Outcome measures of overall physical functioning and quality of life were most frequently being analysed. To a lesser extent, studies applied IRT to PRO measures of specific functioning [27,29-37], pain [35,38-43], psychological constructs [44-46], and work disability [47-51]. Studies also applied IRT to CMs such as measures of disease activity [52-54] and disease damage or radiographic severity [55-57].

### Purpose of analyses

Most common main goals for both the PRO- and the CM-studies were the development or evaluation of new measures, the evaluation of existing measures, and the development or evaluation of alternate or short form versions of an existing measure. In addition, several studies aimed to cross-culturally validate a patient-reported or clinical measure. IRT was rarely applied for the development of item banks [17,58] or computerized adaptive tests [59,60].

### Specific IRT analyses

#### IRT model and software

The vast majority of IRT applications within rheumatology involved Rasch analyses, although a clear specification and rationale of the applied Rasch model was not always given. Few studies used a two-parameter IRT model or Mokken analysis. Most analyses were carried out with the software packages Bigsteps/Winsteps or RUMM.

A motivation of the model choice was only provided in 27.3% of the studies. Likewise, the item and person parameter estimation methods were rarely specified (8.1% and 4.0% of the studies, respectively).

#### IRT assumptions

The assumption of unidimensionality was tested in approximately three quarters of the studies. Methods used for this purpose mainly concerned some type of factor analysis (confirmatory/exploratory factor analysis or principal component analysis) or the examination of specific IRT statistics (e.g. whether the overall model fit or the item fit values were larger than a pre-specified cut-off point). No studies were found where unidimensional IRT models were contrasted with multidimensional IRT models.

A possible violation of the assumption of local independence was evaluated in only one of the CM studies, and in only 18.8% of the studies concerning a PRO. Evaluation of the studies also indicated there was no clear agreement on how to evaluate this assumption, given the variety of methods used.

The assumption of the appropriateness of the model was evaluated by approximately 91% of the studies. When applied, roughly half of the cases evaluated overall fit (PRO: 51.9%, CM: 53.8%), almost all evaluated item fit (PRO: 97.4%, CM: 100.0%), but a much smaller percentage evaluated person fit statistics (PRO: 33.8%, CM: 30.8%).

#### Additional IRT analyses

More than half of the studies used IRT to examine DIF. When applied, analyses varied from cross-sectional DIF across gender (PRO: 80.0%, CM: 66.7%), age (PRO: 76.0%, CM: 66.7%), disease duration (PRO: 36.0%, CM: 16.7), countries/cultures/ethnicity (PRO: 18.0%, CM: 16.7%), and disease type (PRO: 10.0%, CM: 16.7%), to longitudinal DIF analyses over time (PRO: 28.0%, CM: 33.3%).

Other commonly performed IRT analyses included analyses of the global reliability, the hierarchical ordering and distribution of items and persons, and rating scale analyses (i.e. the ordering of the response categories or item thresholds). In addition, a small number of PRO-studies reported IRT analyses regarding the measurement precision of the scale, whereas only 1 of the CM studies evaluated this.

## Discussion

IRT offers a powerful framework for the evaluation or development of existing and new outcome measures. This is the first study that systematically reviewed the extent to which IRT has been applied to measurements from rheumatology. Results showed a marked increase in IRT applications within the rheumatic field from the late eighties up to now. Even though most research focussed on PROs, IRT also appeared to be useful for application to CMs. Some opportunities for further IRT applications and improvements in the analyses and reporting of IRT studies were also pointed out.

IRT can be applied for various purposes. First, IRT analysis is useful for the development and evaluation of new measures [22]. For instance, Helliwell et al. [32] developed a foot impact scale to assess foot status in RA patients. Rasch modeling was used to facilitate item reduction by selecting items which were free of DIF and fitted model expectations. Where the CTT methods often discard items at the extremes of the measurement range because too few patients answer them affirmatively, IRT includes these items since they provide important information at the extremes of the measurement range [61].

IRT is also suitable for the evaluation of existing (ordinal) outcome measures. For example, when evaluating an instrument’s included response categories it can be determined whether they perform as intended or whether categories should be collapsed into fewer options or expanded into more options [22]. Furthermore, it can be evaluated whether the items in the outcome measure form a unidimensional scale as expected or whether item deletion is necessary [22].

Another favourable feature of IRT is that it is expressed at the item level instead of test level as in CTT [11]. By evaluating the performance of individual items, alternate or short form versions of existing measures can be developed. For example, Wolfe et al. [62] developed an alternate version of the HAQ [6,63], known as the HAQ-II, specifically targeted at patients with a relatively high physical functioning.

Another commonly used feature of modeling at the item level is the robust assessment of DIF, as reflected in the high proportion of performed DIF analyses. Nevertheless, the full potential of modeling at the item level is not yet being used, given the low percentage of studies evaluating the items’ performance (i.e. measurement precision and local reliability) along the scale.

When comparing the studies focusing on RA patients with those focusing on OA patients, the measurement intensions of the analysed instruments and the applied IRT models were highly comparable. However, a notable difference was found in the main goals of these studies. Where the RA studies pursued widely varying main goals, including the development of new instruments, the evaluation of existing instruments, the comparison of different instruments, and cross-cultural validation, the studies on OA patients generally focused on the evaluation of existing instruments only.

There are several IRT applications which have not yet been (frequently) used within rheumatology. One IRT application which appears to be still in its infancy within rheumatology, but which is likely to gain importance in the future, is the development of computerized adaptive tests (CATs) [2]. When testing by means of a CAT, every patient receives a test which is tailored (adapted) to his or her level on the underlying construct being measured. Consequently, each patient can be administered different sequences and numbers of items, drawn from a large item bank. By applying CATs, tests can be shortened without any loss of measurement precision, reducing measurement burden for both the patient and the rheumatologist [1,2,9-11,16].

The potential advantages of cross-calibration is another IRT application which has not yet been recognized within rheumatology. As opposed to CTT methods, the item responses are regressed on separate item and person parameters in IRT [11]. This means that the definition of item parameters is independent of the sample receiving the test and the definition of person parameters is independent of the test items given. This separation of parameters facilitates the cross-calibration of various outcome measures based on the same underlying construct [11,64], making their scores comparable with each other.

As discussed earlier, it is important to test the assumptions of unidimensionality, local independence, and model appropriateness when analysing data by means of IRT methods. Items which violate one or more of these assumptions should be combined, rephrased, or deleted [22,23], since they complicate the interpretation of model outcomes. A promising observation was that the majority of the studies tested the assumption of unidimensionality and the appropriateness of the IRT model, albeit some studies did not report any fit statistics. Although comparisons between unidimensional and multidimensional IRT models provide a much more rigorous test of unidimensionality than factor analyses, such comparisons were not made. Analyses of model fit mainly involved overall fit statistics or item fit statistics, and to a lesser extent the evaluation of person fit. Person fit, however, is also important since deviant response patterns of patients may seriously affect the item fit. The removal of patients with such response patterns from the analysis may improve the scale’s internal construct validity significantly [22]. Most studies, however, did not check the assumption of local independence. The importance of local independence has only more recently been recognized and, consequently, only some of the most recent studies (from the year 2007) did evaluate this assumption. Future studies should continue to pay attention to this assumption, since locally dependent items could cause parameter estimates to be biased, which may lead to wrong decisions concerning item selection when constructing a certain outcome measure [15].

The results also showed room for improvement in the reporting of made choices and the rationale for specific decisions. For instance, the applied IRT model is often not specified and, if specified, the reasons behind the selected IRT model and used estimation methods are often not clearly motivated. This complicates the quality appraisal and replication of performed analyses.

Where Belvedere and de Morton [8] examined the application of Rasch analysis only, this study included the whole spectrum of IRT models. A notable finding of this review was that the Rasch models dominate within rheumatology, and that two-parameter IRT models were applied in only a few studies. This may be due to the ease of use of a Rasch model and the easiness with which its results can be interpreted. However, this advantage of Rasch modeling comes with the strict assumption that every item of the measure is equally discriminative. Whether this assumption is appropriate can be tested by comparing the Rasch model fit with the 2-parameter model fit. Since the studies of Pham et al. [65] and Siemons et al. [54] are the only studies in which such a comparison was made, this is a point of interest for future studies.

Although IRT is becoming increasingly popular in health status assessment, IRT is quite complex to understand and is not yet a main-stream technique for most researchers and rheumatologists. To increase common understanding and to improve the interpretation of outcomes resulting from the performed IRT analyses, (bio)statisticians, rheumatologists, and researchers should closely collaborate. Clear guidelines on the quality appraisal of performed IRT analyses might increase the use and understanding of IRT in rheumatology even further. Currently, there are no clear guidelines available for rating the methodological quality of the performed IRT analyses. Although standardized tools like the COSMIN (COnsensusbased Standards for the selection of health status Measurement INstruments) checklist [66] can be used for evaluating the methodological quality of studies on measurement properties, this checklist only contains a few questions regarding IRT analyses and is, therefore, more suitable for analyzing the quality of performed classical test theory analyses. Even though the quality checklist used in this study was based on both expert input and important issues from the literature, it was not exhaustive and, consequently, it might have some limitations. For example, when the sample size was considered, only the absolute number was reported. It was not checked whether the authors also justified the sample size for the analyses they wanted to perform. The varying sample size of the analysed patient groups which was found between studies, might be due to the absence of clear guidelines regarding sample size requirements. It is argued that the most simple Rasch analyses already require a minimum size of 50–100 persons [15,23]. However, many issues are involved in determining the right sample size for a certain study, including the model choice, the number of response categories, and the purpose of the study [15,23]. These issues should be carefully considered to determine the sample size which is minimally needed to achieve reliable model estimates. Consensus and clear guidelines on quality aspects concerning IRT analyses might guide the choice of an adequate sample size and might also stimulate the development of uniform guidelines for performing and reporting IRT studies, and the development of a checklist for evaluating the quality of the performed and reported IRT analyses.

The formulation of such guidelines will provide a strong foundation to future IRT studies. Tennant et al. already provided such guidelines for performing Rasch analyses [22]. However, given the large diversity of approaches, models, and software used in the field of IRT it is difficult to recommend a single set of guidelines for all types of studies, and an expansion or modification of their guidelines might be needed. In order to get sufficient support for these guidelines it is important to first attempt to reach a more global consensus about recommendations. This article could provide input for such attempts and the COSMIN checklist [66] can serve as an example of how such an international approach can lead to the development of a consensus-based checklist. Agreement should be reached on the minimum number of assumptions which should be met (e.g. unidimensionality, model fit, and DIF analysis) and best ways of testing these assumptions. Additionally, this review showed that IRT methods are rarely being applied for the evaluation of an instrument’s local reliability and measurement precision along the scale of the underlying construct and the construction of item banks and CATs, all unique features of IRT. Therefore, it is recommended that more b will be placed on these features in the guidelines and in future studies.

## Conclusions

A marked increase of IRT applications could be observed within rheumatology. IRT has primarily been applied to patient-reported outcomes, but it also appeared to be a useful technique for the evaluation of clinical measures. To date, IRT has mainly been used for the development of new static outcome measures and the evaluation of existing measures. In addition, alternate or short forms were created by evaluating the fit and performance of individual items. Useful IRT applications which are not yet widely used within rheumatology include the cross-calibration of instrument scores and the development of computerized adaptive tests which may reduce the measurement burden for both the patient and the clinician. Also, the measurement precision of outcome measures along the scale has only been evaluated occasionally. The fact that IRT has not yet experienced the same level of standardization and consensus on methodology as CTT methods stresses the importance to adequately explain, justify, and report performed IRT analyses. A global consensus on uniform guidelines should be reached about the minimum number of assumptions which should be met and best ways of testing these assumptions, in order to stimulate the quality appraisal of performed and reported IRT analyses.

## Abbreviations

2-PL model: 2-Parameter Logistic model; CAT: Computerized Adaptive Test; CM: Clinical Measure; CTT: Classical Test Theory; DIF: Differential Item Functioning; HAQ: Health Assessment Questionnaire; IRT: Item Response Theory; OA: OsteoArthritis; PRO: Patient-Reported Outcome; RA: Rheumatoid Arthritis; SF-36: 36-item Short Form health survey.

## Competing interests

The authors declare that they have no competing interests.

## Authors’ contributions

LS was responsible for the conceptualization of the manuscript. LS and PTK were responsible for the screening and identification of studies and the extraction of relevant data. PTK, ET, CG and MVDL supervised the whole study and the interpretation of the results. All authors critically evaluated the manuscript, contributed to its content, and approved the final version.

## Pre-publication history

The pre-publication history for this paper can be accessed here:

http://www.biomedcentral.com/1471-2474/13/216/prepub

## Supplementary Material

Additional file 1 **Checklist.** Both general study information as well as IRT-specific information were extracted, using a purpose-made checklist based on both expert input and important issues as mentioned in Tennant and Conaghan [22], Reeve and Fayers [15], and Orlando [23]. Click here for file

Additional file 2 **List of included articles.** Literature searches in PubMed, Scopus and Web of Science resulted in 99 original English-language articles which used some form of IRT-based analysis of patient reported or clinical outcome data in patients with a rheumatic condition. Click here for file
