# Supplementary material for: Modern psychometrics applied in rheumatology–A systematic review
Source: BMC Musculoskelet Disord. 2012 Oct 31;13:216. doi: 10.1186/1471-2474-13-216 (PMC3517453; doi:10.1186/1471-2474-13-216)
Supplement: Additional file 1 — Checklist. Both general study information as well as IRT-specific information were extracted, using a purpose-made checklist based on both expert input and important issues as mentioned in Tennant and Conaghan [22], Reeve and Fayers [15], and Orlando [23]. [file 1471-2474-13-216-S1.pdf]

List of abbreviations

1-PLM	1-parameter logistic model
2-PLM	2-parameter logistic model
3-PLM	3-parameter logistic model
ADL	Activities of daily living
CAT	Computerized adaptive testing
CFA	Confirmatory factor analysis
CML	Conditional maximum likelihood
DIF	Differential item functioning
EAP	Expected a posteriori estimator
EFA	Exploratory factor analysis
GPCM	Generalized partial credit model
GRM	Graded response model
IADL	Instrumental activities of daily living
IRT	Item response theory
MML	Marginal maximum likelihood
PCA	Principal component analysis
PCM	Partial credit model
PRO	Patient reported outcome
QoL	Quality of life
RSM	Rating scale model
UML	Unconditional maximum likelihood
(W)ML	(Weighted) maximum likelihood

General information

**First author**	…………………………………………………………………………
**Publication year**	☐☐☐☐
**Country / Countries of study execution**	…………………………………………………………………………..
	…………………………………………………………………………..
**Design IRT analyses**	☐ Cross-sectional<br>☐ Longitudinal
**Disease condition(s)**	…………………………………………………………………………
**Total N**	…………………………………………………………………………
**Application**	☐ PRO measure<br>☐ Clinical measure / other

Instrument

Instrument examined ……………………………………………………………………………..........

Main measurement intention

☐ QoL / Health Status

☐ Overall physical function (ADL, IADL, Mobility tasks)

☐ Specific functioning (e.g. hand function)

☐ Disease activity

☐ Pain

☐ Psychosocial construct (e.g. anxiety, depression)

☐ Other: ………………………………………………………………..

Main goal of the study

☐ Development/evaluation of new instrument

☐ Development/evaluation of alternate / short form version

☐ Evaluation of existing instrument

☐ Comparison of instruments

☐ Comparison of the psychometric properties of a single instrument in various patient groups

☐ Scoring of instrument

☐ Cross-calibration / equation of instruments

☐ Calibration / evaluation of item bank

☐ Development / evaluation of CAT

☐ Cross-cultural validation

☐ Other: ………………………………………………………………..

**Applied IRT model**	**General**		**Specific**
	☐ 1-PLM / Rasch	➔	☐ Dichotomous
			☐ Polytomous
			☐ RSM
			☐ PCM
			☐ Other: …………………………………………………
			☐ Not specified
	☐ 2-PLM	➔	☐ Dichotomous
			☐ Polytomous
			☐ GRM
			☐ GPCM
			☐ Other: …………………………………………………
			☐ Not specified
	☐ 3-PLM		
	☐ Mokken		
	☐ Not specified		

Applied IRT software

- ☐ Bigsteps / Winsteps
- ☐ RUMM
- ☐ Multilog / Bilog
- ☐ Parscale
- ☐ MPlus
- ☐ MSP
- ☐ SAS
- ☐ GLAMM / STATA
- ☐ Conquest
- ☐ Other: ………………………………………………………………………………
- ☐ Not specified

Performed analyses

- ☐ Unidimensionality
- ☐ Local independence
- ☐ Item fit
- ☐ Person fit
- ☐ Person/item/subscale separation and/or reliability
- ☐ Measurement precision (e.g. item/test information)
- ☐ DIF
- ☐ Rating scale analysis (response category ordering/item thresholds)
- ☐ Hierarchical ordering and/or distribution of persons/items
- ☐ Cross-calibration / equation
- ☐ Other: ……………………………………………………………………………………

Quality appraisal

**Sample size**	……………………..	
**Number of items**	……………………..	
**Model description**	☐ Yes, with model specification (e.g. Rasch-PCM, Rasch dichotomous)<br>☐ Yes, without model specification<br>☐ No	
**Model choice adequately explained (e.g., number or type of response categories / common discrimination parameter)**	☐ Yes<br>☐ No	
**Applied IRT software is cited**	☐ Yes<br>☐ No	
**Various IRT models were tested**	☐ Yes<br>☐ No	
**Assumptions adequately tested**	☐ Unidimensionality ➔	☐ IRT residuals<br>☐ EFA / PCA of item scores<br>☐ CFA of item scores<br>☐ PCA of residuals<br>☐ IRT statistics (e.g. model fit)<br>☐ Other: ……………………………

☐ Local independence ➔ ☐ IRT residuals
☐ Residual covariation CFA (Modification indices)
☐ Residual covariation PCA / EFA
☐ Other: ……………………………

☐ Fit ➔ ☐ Overall fit statistics
☐ Item fit statistics
☐ Person fit statistics

☐ DIF ➔ ☐ Age
☐ Gender
☐ Disease duration
☐ Countries / cultures
☐ Diseases
☐ Time points
☐ Other: ……………………………

Item parameter estimation method

☐ JML
☐ UML
☐ MML
☐ CML
☐ Bayesian
☐ Not specified

Person/population parameter estimation method

☐ (W)ML
☐ Bayesian (e.g. EAP)
☐ Not specified

Important flaws:

……………………………………………………………………………………………………

……………………………………………………………………………………………………
