# Supplementary material for: Modern psychometrics applied in rheumatology–A systematic review
Source: BMC Musculoskelet Disord. 2012 Oct 31;13:216. doi: 10.1186/1471-2474-13-216 (PMC3517453; doi:10.1186/1471-2474-13-216)
Supplement: Additional file 2 — List of included articles. Literature searches in PubMed, Scopus and Web of Science resulted in 99 original English-language articles which used some form of IRT-based analysis of patient reported or clinical outcome data in patients with a rheumatic condition. [file 1471-2474-13-216-S2.pdf]

Additional file 1

List of included articles

1. Ayis S, Dieppe P: **The natural history of disability and its determinants in adults with lower limb musculoskeletal pain.** *J Rheumatol* 2009, **36:**583-591.

2. Bode RK, Klein-Gitelman MS, Miller ML, Lechman TS, Pachman LM: **Disease activity score for children with juvenile dermatomyositis: Reliability and validity evidence.** *Arthritis Rheum* 2003, **49:**7-15.

3. Boeckstyns MEH: **Development and construct validity of a knee pain questionnaire.** *Pain* 1987, **31:**47-52.

4. Brunner HI, Feldman BM, Urowitz MB, Gladman DD: **Item weightings for the Systemic Lupus International Collaborating Clinics/American College of Rheumatology Disease Damage Index using Rasch analysis do not lead to an important improvement.** *J Rheumatol* 2003, **30:**292-297.

5. Budiman-Mak E, Conrad K, Stuck R, Matters M: **Theoretical model and Rasch analysis to develop a revised Foot Function Index.** *Foot Ankle Int* 2006, **27:**519-527.

6. Chiou CF, Sherbourne CD, Cornelio I, Lubeck DP, Paulus HE, Dylan M, Chang CH, Weisman MH: **Development and validation of the revised Cedars-Sinai health-related quality of life for rheumatoid arthritis instrument.** *Arthritis Rheum* 2006, **55:**856-863.

7. Chiou CF, Sherbourne CD, Ofman J, Lee M, Lubeck DP, Paulus HE, Weisman M: **Development and validation of Cedars-Sinai Health-Related Quality of Life in Rheumatoid Arthritis (CSHQ-RA) short form instrument.** *Arthritis Rheum* 2004, **51:**358-364.

8. Chiou CF, Suarez-Almazor ME, Sherbourne CD, Chang CH, Reyes C, Dylan M, Ofman J, Wallace DJ, Mizutani W, Weisman M: **Development and validation of a preference weight multiattribute health outcome measure for rheumatoid arthritis.** *J Rheumatol* 2006, **33:**2409-2411.

9. Chogle AR, Mistry KJ, Deo SS: **Comparison of the Indian version of Health Assessment Questionnaire Score and Short Form 36 Physical Function Score in rheumatoid arthritis using Rasch analysis.** *Indian J Rheumatol* 2008, **3:**52-57.

10. Cieza A, Hilfiker R, Boonen A, Chatterji S, Kostanjsek N, Ustun BT, Stucki G: **Items from patient-oriented instruments can be integrated into interval scales to operationalize categories of the International Classification of Functioning, Disability and Health.** *J Clin Epidemiol* 2009, **62:**912-921.

11. Cieza A, Hilfiker R, Boonen A, van der Heijde D, Braun J, Stucki G: **Towards an ICF-based clinical measure of functioning in people with ankylosing spondylitis: A methodological exploration.** *Disabil Rehabil* 2009, **31:**528-537.

12. Conaghan PG, Emerton M, Tennant A: **Internal construct validity of the Oxford knee scale: Evidence from Rasch measurement.** *Arthritis Care Res* 2007, **57:**1363-1367.

13. Conaghan PG, Tennant A, Peterfy CG, Woodworth T, Stevens R, Guermazi A, Genant H, Felson DT, Hunter D: **Examining a whole-organ magnetic resonance imaging scoring system for osteoarthritis of the knee using Rasch analysis.** *Osteoarthritis Cartilage* 2006, **14 Suppl A:**A116-121.

14. Covic T, Pallant JF, Conaghan PG, Tennant A: **A longitudinal evaluation of the Center for Epidemiologic Studies-Depression scale (CES-D) in a rheumatoid arthritis population using Rasch analysis.** *Health Qual Life Outcomes* 2007, **5:**41.

15. Covic T, Pallant JF, Tennant A, Cox S, Emery P, Conaghan PG: **Variability in depression prevalence in early rheumatoid arthritis: a comparison of the CES-D and HAD-D Scales.** *BMC Musculoskelet Disord* 2009, **10:**18.

16. Davis AM, Badley EM, Beaton DE, Kopec J, Wright JG, Young NL, Williams JI: **Rasch analysis of the Western Ontario McMaster (WOMAC) Osteoarthritis Index: results from community and arthroplasty samples.** *J Clin Epidemiol* 2003, **56:**1076-1083.

17. Davis AM, Perruccio AV, Canizares M, Tennant A, Hawker GA, Conaghan PG, Roos EM, Jordan JM, Maillefert JF, Dougados M, Lohmander LS: **The development of a short measure of physical function for hip OA HOOS-Physical Function Shortform (HOOS-PS): an OARSI/OMERACT initiative.** *Osteoarthritis Cartilage* 2008, **16:**551-559.

18. Doward LC, McKenna SP, Meads DM, Twiss J, Revicki D, Wong RL, Luo MP: **Translation and validation of non-English versions of the Ankylosing Spondylitis Quality of Life (ASQOL) questionnaire.** *Health Qual Life Outcomes* 2007, **5**.

19. Doward LC, McKenna SP, Whalley D, Tennant A, Griffiths B, Emery P, Veale DJ: **The development of the L-QoL: a quality-of-life instrument specific to systemic lupus erythematosus.** *Ann Rheum Dis* 2009, **68:**196-200.

20. Doward LC, Spoorenberg A, Cook SA, Whalley D, Helliwell PS, Kay LJ, McKenna SP, Tennant A, Van der Heijde D, Chamberlain MA: **Development of the ASQoL: A quality of life instrument specific to ankylosing spondylitis.** *Ann Rheum Dis* 2003, **62:**20-26.

21. Durez P, Fraselle V, Houssiau F, Thonnard JL, Nielens H, Penta M: **Validation of the ABILHAND questionnaire as a measure of manual ability in patients with rheumatoid arthritis.** *Ann Rheum Dis* 2007, **66:**1098-1105.

22. El Miedany Y, El Gaafary M, El Aroussy N, Ahmed I, Youssef S, Palmer D: **Patient reported outcomes in ankylosing spondylitis: development and validation of a new questionnaire for functional impairment and quality of life assessment.** *Clin Exp Rheumatol* 2011, **29:**801-810.

23. Eyres S, Tennant A, Kay L, Waxman R, Helliwell PS: **Measuring disability in ankylosing spondylitis: comparison of bath ankylosing spondylitis functional index with revised Leeds Disability Questionnaire.** *J Rheumatol* 2002, **29:**979-986.

24. Gilworth G, Chamberlain MA, Bhakta B, Haskard D, Silman A, Tennant A: **Development of the BD-QoL: a quality of life measure specific to Behcet's disease.** *J Rheumatol* 2004, **31:**931-937.

25. Gilworth G, Chamberlain MA, Harvey A, Woodhouse A, Smith J, Smyth MG, Tennant A: **Development of a work instability scale for rheumatoid arthritis.** *Arthritis Rheum* 2003, **49:**349-354.

26. Gilworth G, Emery P, Barkham N, Smyth MG, Helliwell P, Tennant A: **Reducing work disability in Ankylosing Spondylitis: development of a work instability scale for AS.** *BMC Musculoskelet Disord* 2009, **10:**68.

27. Gilworth G, Emery P, Gossec L, Vliet Vlieland TP, Breedveld FC, Hueber AJ, Schett G, Tennant A: **Adaptation and cross-cultural validation of the rheumatoid arthritis work instability scale (RA-WIS).** *Ann Rheum Dis* 2009, **68:**1686-1690.

28. Goetz C, Ecosse E, Rat AC, Pouchot J, Coste J, Guillemin F: **Measurement properties of the osteoarthritis of knee and hip quality of life OAKHQOL questionnaire: an item response theory analysis.** *Rheumatology* 2011, **50:**500-505.

29. Haley SM, McHorney CA, Ware JE: **Evaluation of the MOS SF-36 physical functioning scale (PF-10): I. Unidimensionality and reproducibility of the Rasch item scale.** *J Clin Epidemiol* 1994, **47:**671-684.

30. Hassett AL, Li T, Buyske S, Savage SV, Gignac MA: **The multi-faceted assessment of independence in patients with rheumatoid arthritis: preliminary validation from the ATTAIN study.** *Curr Med Res Opin* 2008, **24:**1443-1453.

31. Haugen IK, Moe RH, Slatkowsky-Christensen B, Kvien TK, van der Heijde D, Garratt A: **The AUSCAN subscales, AIMS-2 hand/finger subscale, and FIOHA were not unidimensional scales.** *J Clin Epidemiol* 2011, **64:**1039-1046.

32. Haywood KL, Garratt AM, Jordan KP, Healey EL, Packham JC: **Evaluation of ankylosing spondylitis quality of life (EASi-QoL): Reliability and validity of a new patient-reported outcome measure.** *J Rheumatol* 2010, **37:**2100-2109.

33. Helliwell P, Reay N, Gilworth G, Redmond A, Slade A, Tennant A, Woodburn J: **Development of a foot impact scale for rheumatoid arthritis.** *Arthritis Rheum* 2005, **53:**418-422.

34. Hirsch JD, Lee SJ, Terkeltaub R, Khanna D, Singh J, Sarkin A, Harvey J, Kavanaugh A: **Evaluation of an Instrument Assessing Influence of Gout on Health-Related Quality of Life.** *J Rheumatol* 2008, **35:**2406-2414.

35. Jette AM, McDonough CM, Haley SM, Ni PS, Olarsch S, Latham N, Hambleton RK, Felson D, Kim YJ, Hunter D: **A computer-adaptive disability instrument for lower extremity osteoarthritis research demonstrated promising breadth, precision, and reliability.** *J Clin Epidemiol* 2009, **62:**807-815.

36. Katz PP, Radvanski DC, Allen D, Buyske S, Schiff S, Nadkarni A, Rosenblatt L, Maclean R, Hassett AL: **Development and validation of a short form of the valued life activities disability questionnaire for rheumatoid arthritis.** *Arthritis Care Res* 2011, **63:**1664-1671.

37. Keenan AM, McKenna SP, Doward LC, Conaghan PG, Emery P, Tennant A: **Development and validation of a needs-based quality of life instrument for osteoarthritis.** *Arthritis Care Res* 2008, **59:**841-848.

38. Kelly PA, Kallen MA, Suarez-Almazor ME: **A combined-method psychometric analysis recommended modification of the multidimensional health locus of control scales.** *J Clin Epidemiol* 2007, **60:**440-447.

39. Kersten P, White PJ, Tennant A: **The Visual Analogue WOMAC 3.0 scale - internal validity and responsiveness of the VAS version.** *BMC Musculoskelet Disord* 2010, **11**.

40. Ko Y, Lo N-N, Yeo S-J, Yang K-Y, Yeo W, Chong H-C, Thumboo J: **Rasch analysis of the Oxford Knee Score.** *Osteoarthr Cartilage* 2009, **17:**1163-1169.

41. Kopec JA, Sayre EC, Davis AM, Badley EM, Abrahamowicz M, Sherlock L, Williams JI, Anis AH, Esdaile JM: **Assessment of health-related quality of life in arthritis: conceptualization and development of five item banks using item response theory.** *Health Qual Life Outcomes* 2006, **4:**33.

42. Köse SK, Öztuna D, Kutlay S, Elhan AH, Tennant A, Kücükdeveci AA: **Psychometric properties of the Health Assessment Questionnaire Disability Index (HAQ-DI) and the Modified Health Assessment Questionnaire (MHAQ) in patients with knee osteoarthritis.** *Turk J Rheumatol* 2010, **25:**147-155.

43. Kosinski M, Bjorner JB, Ware JE, Sullivan E, Straus WL: **An evaluation of a patient-reported outcomes found computerized adaptive testing was efficient in assessing osteoarthritis impact.** *J Clin Epidemiol* 2006, **59:**715-723.

44. Kristjansson E, Tugwell PS, Wilson AJ, Brooks PM, Driedger SM, Gallois C, O'Connor AM, Qualman A, Santesso N, Wale J, Wells GA: **Development of the effective musculoskeletal consumer scale.** *J Rheumatol* 2007, **34:**1392-1400.

45. Kucukdeveci AA, Sahin H, Ataman S, Griffiths B, Tennant A: **Issues in cross-cultural validity: example from the adaptation, reliability, and validity testing of a Turkish version of the Stanford Health Assessment Questionnaire.** *Arthritis Rheum* 2004, **51:**14-19.

46. Kurtais Y, Oztuna D, Küçükdeveci AA, Kutlay S, Hafiz M, Tennant A: **Reliability, construct validity and measurement potential of the ICF comprehensive core set for osteoarthritis.** *Bmc Musculoskel Dis* 2011, **12:**255.

47. Kutlay S, Kucukdeveci AA, Gonul D, Tennant A: **Adaptation and validation of the Turkish version of the Rheumatoid Arthritis Quality of Life Scale.** *Rheumatol Int* 2003, **23:**21-26.

48. Lawton G, Bhakta BB, Chamberlain MA, Tennant A: **The Behcet's disease activity index.** *Rheumatology* 2004, **43:**73-78.

49. Lee YS, Douglas J, Chewning B: **Techniques for developing health quality of life scales for point of service use.** *Soc Indic Res* 2007, **83:**331-350.

50. Leong KP, Kong KO, Thong BY, Koh ET, Lian TY, Teh CL, Cheng YK, Chng HH, Badsha H, Law WG, et al: **Development and preliminary validation of a systemic lupus erythematosus-specific quality-of-life instrument (SLEQOL).** *Rheumatology* 2005, **44:**1267-1276.

51. Leung YY, Tam LS, Kun EW, Ho KW, Li EK: **Comparison of 4 functional indexes in psoriatic arthritis with axial or peripheral disease subgroups using Rasch analyses.** *J Rheumatol* 2008, **35:**1613-1621.

52. Martin M, Kosinski M, Bjorner JB, Ware JE, Jr., Maclean R, Li T: **Item response theory methods can improve the measurement of physical function by combining the modified health assessment questionnaire and the SF-36 physical function scale.** *Qual Life Res* 2007, **16:**647-660.

53. McHorney CA, Cohen AS: **Equating health status measures with item response theory.** *Med Care* 2000, **38:**II-43-II-59.

54. McKenna SP, Doward LC, Whalley D, Tennant A, Emery P, Veale DJ: **Development of the PsAQoL: a quality of life instrument specific to psoriatic arthritis.** *Ann Rheum Dis* 2004, **63:**162-169.

55. McTaggart-Cowan HM, Brazier JE, Tsuchiya A: **Clustering Rasch results: A novel method for developing rheumatoid arthritis states for use in valuation studies.** *Value Health* 2010, **13:**787-795.

56. Mielenz TJ, Edwards MC, Callahan LF: **First item response theory analysis on Tampa Scale for Kinesiophobia (fear of movement) in arthritis.** *J Clin Epidemiol* 2010, **63:**315-320.

57. Mielenz TJ, Edwards MC, Callahan LF: **Item-response-theory analysis of two scales for self-efficacy for exercise behavior in people with arthritis.** *J Aging Phys Act* 2011, **19:**239-248.

58. Moorer P, Suurmeije Th P, Foets M, Molenaar IW: **Psychometric properties of the RAND-36 among three chronic diseases (multiple sclerosis, rheumatic diseases and COPD) in The Netherlands.** *Qual Life Res* 2001, **10:**637-645.

59. Ndosi M, Tennant A, Bergsten U, Kukkurainen ML, Machado P, de la Torre-Aboki J, Vliet Vlieland TP, Zangi HA, Hill J: **Cross-cultural validation of the Educational Needs Assessment Tool in RA in 7 European countries.** *BMC Musculoskelet Disord* 2011, **12:**110.

60. Niedermann K, Forster A, Ciurea A, Hammond A, Uebelhart D, de Bie R: **Development and psychometric properties of a joint protection self-efficacy scale.** *Scand J Occup Ther* 2011, **18:**143-152.

61. Niedermann K, Forster A, Hammond A, Uebelhart D, de Bie R: **Development and validation of a German version of the joint protection behavior assessment in patients with rheumatoid arthritis.** *Arthritis Rheum* 2007, **57:**249-255.

62. Nordenskiold U, Grimby G, Hedberg M, Wright B, Linacre JM: **The structure of an instrument for assessing the effects of assistive devices and altered working methods in women with rheumatoid arthritis.** *Arthritis Care Res* 1996, **9:**358-367.

63. O'Malley KJ, Suarez-Almazor M, Aniol J, Richardson P, Kuykendall DH, Moseley JB, Jr., Wray NP: **Joint-specific multidimensional assessment of pain (J-MAP): factor structure, reliability, validity, and responsiveness in patients with knee osteoarthritis.** *J Rheumatol* 2003, **30:**534-543.

64. Osborne RH, Elsworth GR, Whitfield K: **The Health Education Impact Questionnaire (heiQ): an outcomes and evaluation measure for patient education and self-management interventions for people with chronic conditions.** *Patient Educ Couns* 2007, **66:**192-201.

65. Pallant JF, Keenan AM, Misajon R, Conaghan PG, Tennant A: **Measuring the impact and distress of osteoarthritis from the patients' perspective.** *Health Qual Life Outcomes* 2009, **7.**

66. Paulsen T, Grotle M, Garratt A, Kjeken I: **Development and psychometric testing of the patient-reported measure of activity performance of the hand (MAP-Hand) in rheumatoid arthritis.** *J Rehabil Med* 2010, **42:**636-644.

67. Penta M, Thonnard JL, Tesio L: **ABILHAND: a Rasch-built measure of manual ability.** *Arch Phys Med Rehabil* 1998, **79:**1038-1042.

68. Perkins K, Hoffman RW, Bezruczko N: **A Rasch analysis for classification of systemic lupus erythematosus and mixed connective tissue disease.** *J Appl Meas* 2008, **9:**136-150.

69. Perruccio AV, Lohmander LS, Canizares M, Tennant A, Hawker GA, Conaghan PG, Roos EM, Jordan JM, Maillefert JF, Dougados M, Davis AM: **The development of a short measure of physical function for knee OA KOOS-Physical Function Shortform (KOOS-PS) - an OARSI/OMERACT initiative.** *Osteoarthritis Cartilage* 2008, **16:**542-550.

70. Pham T, van der Heijde DM, Pouchot J, Guillemin F: **Development and validation of the French ASQoL questionnaire.** *Clin Exp Rheumatol* 2010, **28:**379-385.

71. Pollard B, Dixon D, Dieppe P, Johnston M: **Measuring the ICF components of impairment, activity limitation and participation restriction: an item analysis using classical test theory and item response theory.** *Health Qual Life Outcomes* 2009, **7:**41.

72. Pouchot J, Ecosse E, Coste J, Guillemin F: **Validity of the childhood health assessment questionnaire is independent of age in juvenile idiopathic arthritis.** *Arthritis Rheum* 2004, **51:**519-526.

73. Rauch A, Cieza A, Boonen A, Ewert T, Stucki G: **Identification of similarities and differences in functioning in persons with rheumatoid arthritis and ankylosing spondylitis using the International Classification of Functioning, Disability and Health (ICF).** *Clin Exp Rheumatol* 2009, **27:**S92-101.

74. Roorda LD, Jones CA, Waltz M, Lankhorst GJ, Bouter LM, van der Eijken JW, Willems WJ, Heyligers IC, Voaklander DC, Kelly KD, Suarez-Almazor ME: **Satisfactory cross cultural equivalence of the Dutch WOMAC in patients with hip osteoarthritis waiting for arthroplasty.** *Ann Rheum Dis* 2004, **63:**36-42.

75. Ryser L, Wright BD, Aeschlimann A, Mariacher-Gehler S, Stucki G: **A new look at the Western Ontario and McMaster Universities Osteoarthritis Index using Rasch analysis.** *Arthritis Care Res* 1999, **12:**331-335.

76. Sheehan TJ, DeChello LM, Garcia R, Fifield J, Rothfield N, Reisine S: **Measuring disability: application of the Rasch model to activities of daily living (ADL/IADL).** *J Outcome Meas* 2001, **5:**839-863.

77. Sheehan TJ, DuBrava S, Fifield J, Reisine S, DeChello L: **Rate of change in functional limitations for patients with rheumatoid arthritis: effects of sex, age, and duration of illness.** *J Rheumatol* 2004, **31:**1286-1292.

78. Siemons L, ten Klooster PM, Taal E, Kuper IH, van Riel P, van de Laar M, Glas CAW: **Validating the 28-Tender Joint Count Using Item Response Theory.** *J Rheumatol* 2011, **38:**2557-2564.

79. Steultjens MPM, Dekker J, van Baar ME, Oostendorp RAB, Bijlsma JWJ: **Internal consistency and validity of an observational method for assessing disability in mobility in patients with osteoarthritis.** *Arthritis Care Res* 1999, **12:**19-25.

80. Suurmeijer TP, Doeglas DM, Moum T, Briancon S, Krol B, Sanderman R, Guillemin F, Bjelle A, van den Heuvel WJ: **The Groningen Activity Restriction Scale for measuring disability: its utility in international comparisons.** *Am J Public Health* 1994, **84:**1270-1273.

81. Tammaru M, McKenna SP, Meads DM, Maimets K, Hansen E: **Adaptation of the rheumatoid arthritis quality of life scale for Estonia.** *Rheumatol Int* 2006, **26:**655-662.

82. Tang K: **Disease-related differential item functioning in the work instability scale for rheumatoid arthritis: converging results from three methods.** *Arthritis Care Res* 2011, **63:**1159-1169.

83. Tang K, Beaton DE, Lacaille D, Gignac MAM, Zhang W, Anis AH, Bombardier C, Canadian Arthrit Network Work P: **The Work Instability Scale for Rheumatoid Arthritis (RA-WIS): Does it work in osteoarthritis?** *Qual Life Res* 2010, **19:**1057-1068.

84. Taylor WJ, Colvine K, Gregory K, Collis J, McQueen FM, Dalbeth N: **The Health Assessment Questionnaire Disabillity Index is a valid measure of physical function in gout.** *Clin Exp Rheumatol* 2008, **26:**620-626.

85. Taylor WJ, McPherson KM: **Using Rasch analysis to compare the psychometric properties of the Short Form 36 physical function score and the Health Assessment Questionnaire disability index in patients with psoriatic arthritis and rheumatoid arthritis.** *Arthritis Rheum* 2007, **57:**723-729.

86. ten Klooster PM, Taal E, van de Laar MA: **Rasch analysis of the Dutch Health Assessment Questionnaire disability index and the Health Assessment Questionnaire II in patients with rheumatoid arthritis.** *Arthritis Rheum* 2008, **59:**1721-1728.

87. Tennant A, Hillman M, Fear J, Pickering A, Chamberlain MA: **Are we making the most of the Stanford Health Assessment Questionnaire?** *Br J Rheumatol* 1996, **35:**574-578.

88. Tennant A, Kearns S, Turner F, Wyatt S, Haigh R, Chamberlain MA: **Measuring the function of children with juvenile arthritis.** *Rheumatology* 2001, **40:**1274-1278.

89. Uhlig T, Lillemo S, Moe RH, Stamm T, Cieza A, Boonen A, Mowinckel P, Kvien TK, Stucki G: **Reliability of the ICF Core Set for rheumatoid arthritis.** *Ann Rheum Dis* 2007, **66:**1078-1084.

90. van Groen MM, ten Klooster PM, Taal E, van de Laar MAFJ, Glas CAW: **Application of the health assessment questionnaire disability index to various rheumatic diseases.** *Qual Life Res* 2010, **19:**1255-1263.

91. Vanthuyne M, Smith V, Arat S, Westhovens R, Keyser FD, Houssiau FA, Thonnard JL, Vandervelde L: **Validation of a manual ability questionnaire in patients with systemic sclerosis.** *Arthritis Care Res* 2009, **61:**695-703.

92. Wolfe F: **Which HAQ is best? A comparison of the HAQ, MHAQ and RA-HAQ, a difficult 8 item HAQ (DHAQ), and a rescored 20 item HAQ (HAQ20): analyses in 2,491 rheumatoid arthritis patients following leflunomide initiation.** *J Rheumatol* 2001, **28:**982-989.

93. Wolfe F: **Pain extent and diagnosis: development and validation of the regional pain scale in 12,799 patients with rheumatic disease.** *J Rheumatol* 2003, **30:**369-378.

94. Wolfe F, Hawley DJ, Goldenberg DL, Russell IJ, Buskila D, Neumann L: **The assessment of functional impairment in fibromyalgia (FM): Rasch analyses of 5 functional scales and the development of the FM Health Assessment Questionnaire.** *J Rheumatol* 2000, **27:**1989-1999.

95. Wolfe F, Kong SX: **Rasch analysis of the Western Ontario MacMaster questionnaire (WOMAC) in 2205 patients with osteoarthritis, rheumatoid arthritis, and fibromyalgia.** *Ann Rheum Dis* 1999, **58:**563-568.

96. Wolfe F, Michaud K, Kahler K, Omar M: **The Short Arthritis Assessment Scale: a brief assessment questionnaire for rapid evaluation of arthritis severity in research and clinical practice.** *J Rheumatol* 2004, **31:**2472-2479.

97. Wolfe F, Michaud K, Pincus T: **Development and validation of the health assessment questionnaire II: a revised version of the health assessment questionnaire.** *Arthritis Rheum* 2004, **50:**3296-3305.

98. Wolfe F, van der Heijde DM, Larsen A: **Assessing radiographic status of rheumatoid arthritis: introduction of a short erosion scale.** *J Rheumatol* 2000, **27:**2090-2099.

99. Woodburn J, Vliet Vlieland TP, van der Leeden M, Steultjens MP: **Rasch analysis of Dutch-translated version of the Foot Impact Scale for rheumatoid arthritis.** *Rheumatology* 2011, **50:**1315-1319.
